# The impact of CoronaVac on the neutralization breadth and magnitude of the antibody response to SARS-CoV-2 viruses

**DOI:** 10.3389/fimmu.2022.990071

**Published:** 2022-09-20

**Authors:** Lu Zhang, Hongquan Chen, Su Yang, Yang Zhao, Xiaoyun Shen, Xiaowen He, Haohui Ye, Deqin Wang, Jiazhou Lou, Yinshan Wang, Shengjun Wu

**Affiliations:** ^1^ Department of Clinical Laboratory, Sir Run Run Shaw Hospital, Zhejiang University School of Medicine, Hangzhou, China; ^2^ Key Laboratory of Precision Medicine in Diagnosis and Monitoring Research of Zhejiang Province, Hangzhou, China; ^3^ Key Laboratory of Endoscopic Technology Research, Sir Run Run Shaw Hospital, Zhejiang University School of Medicine, Hangzhou, China; ^4^ Health Care Department, Sir Run Run Shaw Hospital, Zhejiang University School of Medicine, Hangzhou, China

**Keywords:** SARS-CoV-2, booster vaccination, neutralization breadth, variants, Omicron

## Abstract

Although immune response enhancement has been reported after primary and booster vaccines of CoronaVac, neutralization breadth of SARS-CoV-2 variants is still unclear. In the present study, we examined the neutralization magnitude and breadth of SARS-CoV-2 variants including Beta (B.1.351), Delta (B.1.617.2) and Omicron (B.1.1.529) in 33 convalescent COVID-19 patients and a cohort of 55 medical staff receiving primary CoronaVac vaccines and an additional homologous booster dose. Results showed that, as compared with the two-dose primary vaccination, the homologous booster dose achieved 2.24-, 3.98-, 4.58- and 2.90-fold increase in neutralization titer against wild-type, Beta, Delta, and Omicron, respectively. After booster dose, neutralization titer reduction for variants was less than that after the primary vaccine or that for convalescents. The proportion of recipients able to neutralize 2 or more variants increased from 36.36% post the primary vaccination to 87.27% after the booster. Significant increase in neutralization breadth of 1.24 (95% confidence interval (CI), 0.89–1.59) variants was associated with a log_10_ increase in neutralization titer against the wild-type. In addition, anti-RBD IgG level was identified as an excellent surrogate for positive neutralization of SARS-CoV-2 and neutralization breadth of variants. These findings highlight the value of an additional homologous CoronaVac dose in broadening the cross-neutralization against SARS-CoV-2 variants, and are critical for informing the booster dose vaccination efforts.

## Introduction

Since the emerging of severe acute respiratory syndrome coronavirus 2 (SARS-CoV-2) in December, 2019, over 496 million confirmed cases and over 6 million deaths have been reported globally, as of 10 April 2022 (1). Until now, a variety of SARS-CoV-2 variants with greater transmissibility or immune evasion have been found including Beta, Delta, Omicron (2). The Beta and Delta variants exhibited a lower neutralizing sensitivity to immune sera elicited by vaccination or prior infection (3, 4). The Omicron variant carrying more than 30 mutations in its spike protein was firstly identified in South Africa, and has now spread rapidly internationally (5). Therefore, concerns have been raised whether or to what degree will the emerging variants escape the existing immunity.

At present, several vaccines with different technical routes, including RNA-based vaccines (e.g. BNT162b2 and mRNA-1273), non-replicating viral vector vaccines (e.g. AZD1222), and inactivated whole-virion vaccines (e.g. CoronaVac and BBIBP-CorV), have been widely used in the world. A few studies have reported the immunogenicity and effectiveness of these vaccines against wild-type SARS-CoV-2 and circulating variants. Neutralizing titers against wild-type SARS-CoV-2 were about 300-2000 by BNT162b2 (6–10), 300-5000 by mRNA-1273 (4, 11–14), 150-500 by AZD1222 (15–17), and 50-200 by inactivated vaccines (18–21) one week to one month post the second dose, while neutralizing capability against variants significantly decreased as compared with that against wild-type (20–25). Coronavac is one of the most widely used inactivated vaccines globally, which is proved to induce effective immune responses among recipients (26). A large phase 3 trial of CoronaVac in Brazil demonstrated that two doses, administered at an interval of 14 days, had an efficacy of 51% against symptomatic infection, 100% against severe COVID-19, and 100% against hospitalization (27). However, a few studies have shown that two-dose CoronaVac vaccination elicited limited neutralizing capability against variants especially Omicron (28, 29). Therefore, additional vaccination booster dose is recommended to restore vaccine effectiveness (30). Although booster vaccination is believed to enhance immune responses, limited information is available whether it broadens the breadth of protection against variants.

In the present study, we aimed to assess the neutralization responses in convalescent COVID-19 patients not vaccinated as well as a cohort of medical staff receiving primary and booster CoronaVac vaccinations. We analysed antibody dynamics and neutralization breadth of variants including Beta, Delta and Omicron after receiving a primary vaccination and an additional homologous booster dose. We also assessed the performance of anti-receptor binding domain (anti-RBD) IgG level as a surrogate for variants neutralization.

## Materials and methods

### Participants and blood sampling

**Figure 1:**
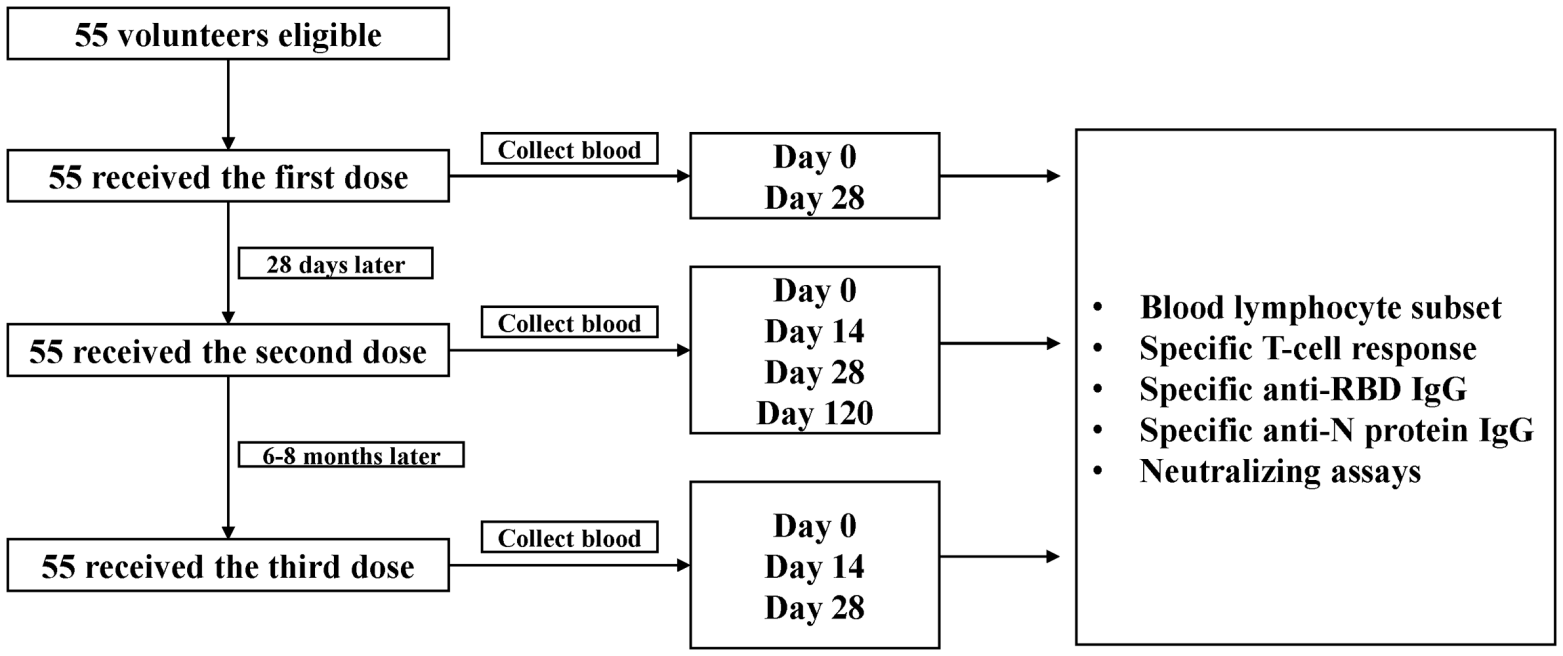
Study flowchart.

A cohort of 55 medical staff was established in Sir Run Run Shaw Hospital, Zhejiang University School of Medicine, Hangzhou, China, to explore the dynamics of neutralization antibody responses against SARS-CoV-2 and variants after receiving CoronaVac vaccine (Sinovac Biotech Ltd., Beijing, China). Healthy adults ≥18 years who tested negative for SARS-CoV-2 RNA, anti-SARS-CoV-2 IgM or IgG were eligible for vaccination and enrollment in the study. Viral RNA was detected from pharyngeal swabs or sputum using a commercial quantitative real-time RT-PCR assay (BioGerm Medical Technology Co. Ltd., Shanghai, China). Specific anti-SARS-CoV-2 IgM and IgG in serum were tested using a commercial chemiluminescence immunoassay (CLIA) (YHLO, Shenzhen, Guangdong, China). Participants with a history of allergy to any ingredient included in the vaccines, acute febrile disease on the day of injection, pregnancy, epilepsy or other uncontrolled diseases of nervous system, immune deficiency or using immunosuppressive drugs, or hemorrhagic diseases were excluded. Participants were scheduled to receive the primary 2 doses with an interval of 4 weeks, and an additional homologous booster dose six to eight months later (**Figure 1**).

Blood samples of medical staff were collected on day 0 and day 28 after the first dose, day 14, day 28 and day 120 after the second dose, and day 0, day 14 and day 28 after the third dose (booster dose). T-cell responses and blood lymphocyte subset distribution were assessed immediately using the freshly collected peripheral blood mononuclear cells. The serum samples were stored as aliquots at -80°C until analysis.

Archived serum samples from 33 not vaccinated convalescent COVID-19 patients were also applied for neutralization assays. The 33 patients were diagnosed with COVID-19 based on SARS-CoV-2 RNA positive testing and chest CT following symptom onset between January 31 and February 20, 2020. These patients originated from a prison transmission cluster. The serum samples were collected and frozen at -80°C on April 27, 2020, when they were tested SARS-CoV-2 RNA negative. This study was approved by the Medical Ethical Committee of Sir Run Run Shaw Hospital (Scientific Research 20210210-236). Written informed consents were obtained from all participants.

### Assessment of vaccination safety

A specific adverse event card was designed to record solicited systemic and local adverse reactions. The event cards were filled by participants and collected by trained personnel on day 0, day 14 and day 28 after the second and third dose injection.

### Serum pseudovirus neutralization test (pVNT)

ACE2-high-expressed 293T cells (HEK293T) and lentivirus-based SARS-CoV-2 pseudoviruses incorporating with spike protein from the wild-type (Wuhan-1 reference strain) and variants of Beta (B.1.351), Delta (B.1.617.2), and Omicron (B.1.1.529) were purchased from GenScript (GenScript, Nanjing, Jiangsu, China). Pseudoviruses were constructed by co-transfection of an env-expressing plasmid for respective SARS-CoV-2 spike protein and a HIV-1 backbone plasmid expressing packaging proteins and luciferase reporter in HEK293T cells. All serum samples were tested after inactivation at 56°C for 30 min. Briefly, 25 μL of pseudovirus (3×10^5^ TCID50/mL) and 25 μL of serial dilutions of serum or positive control monoclonal antibody against ACE2 (10-fold initial dilution, followed by seven serial 4-fold dilutions) (L02087A, GensSript, Nanjing, Jiangsu, China) were mixed and incubated at room temperature for 1 h. Then, the 50 μL mixtures were added into each well of a 96-well plate as well as 50 μL HEK293T cells (6 × 10^4^ cells/mL). After culture at 37°C in a humidified atmosphere with 5% CO_2_ for 24 h, additional 50 μL of fresh DMEM complete medium (Gibco, Shanghai, China) were added. Additional 24 h later, the luminescence was measured using Fire-Lumi™ (GenScript, Nanjing, China) and detected for relative light units (RLUs) using SpectraMax M5^e^ (Molecular Devices, San Jose, CA, USA). The pVNT titer was determined by the half-maximal inhibitory concentration (IC50) calculated using nonlinear regression in GraphPad Prism. IC50 <10 was set as the detection limit of neutralization.

### T-cell responses assessment

An interferon (IFN) γ enzyme-linked immunospot (ELISpot) assay (Dayou, Shenzhen, China) was used to quantify specific T-cell responses. Fresh peripheral blood mononuclear cells were purified and stimulated with SARS-CoV-2 spike (S) protein, nucleocapsid (N) protein, membrane (M) protein, open reading frame (O) protein defined peptide pool, which contains 47 synthetic overlapping peptides (Mabtech AB, Stockholm, Sweden), for about 12-24 h before detection. Results were expressed as the number of spot-forming cells per 2 × 10^5^ cells.

### Specific IgG antibody testing

Specific IgG antibodies against RBD (anti-RBD IgG) and nucleocapsid protein of SARS-CoV-2 (anti-NP IgG) were detected using Axceed 360/COVID-19-IgM-IgG (Bioscience, Tianjin, China) and Wan 200+/2019-nCoV Ab CLIA assays (Wantai BioPharm, Beijing, China), respectively. Results were expressed as cut off index (COI) and a value lower than 1 was considered negative. COI values were used to estimate the response level.

### Statistical analysis

For most variables, we calculated descriptive statistics, such as medians with interquartile ranges (IQR) and proportions. Geometric mean titer (GMT) with 95% confidence interval (CI) were calculated for log-transformed pVNT titers. Statistical comparisons between groups were evaluated by Wilcoxon signed-ranked test. Multivariate regression analysis was conducted to identify correlates of breadth of neutralization antibodies. Receiver operating characteristic (ROC) analyses were conducted to assess the ability of anti-RBD IgG level to predict neutralization titer against wild-type, Beta, Delta or Omicron pseudoviruses (titer ≥10: positive; titer <10: negative), and the neutralization breadth of variants (breadth ≥2 variants neutralized: broad; breadth <2: limited). All statistical analyses were performed using R, version 3.4.3 (R Project for Statistical Computing) and GraphPad Prism 9.1.1 (GraphPad Software, San Diego, CA, USA). A p-value less than 0.05 was considered significant. One star (*) is used if the p-value is less than 0.05, two stars (**) represent p-values less than 0.05 but higher or equal to 0.001, and three stars (***) show a p-value below 0.001.

## Results

### Participants

A total of 55 participants receiving both primary and booster doses of CoronaVac were enrolled. The participants had a median age of 29 years (IQR, 23-45), with 56.36% female and median BMI of 20.91 kg/m^2^ (IQR, 18.29-24.11) (**Table 1**). Both the primary and booster CoronaVac vaccinations were well tolerated among recipients (**Table S1**).

**Table 1:** Baseline characteristics of vaccine recipients.

Characteristics	CoronaVac recipients (n=55)
Age (year)	29 (23-45)
Female	31 (56.36%)
Body mass index (kg/m^2^)	20.91 (18.29-24.11)

Data expressed as median (IQR) or number (percentage) of vaccine recipients.

### Neutralization of the Beta, Delta and Omicron SARS-CoV-2 variants is impaired in convalescent patients and primary vaccine recipients

**Figure 2:**
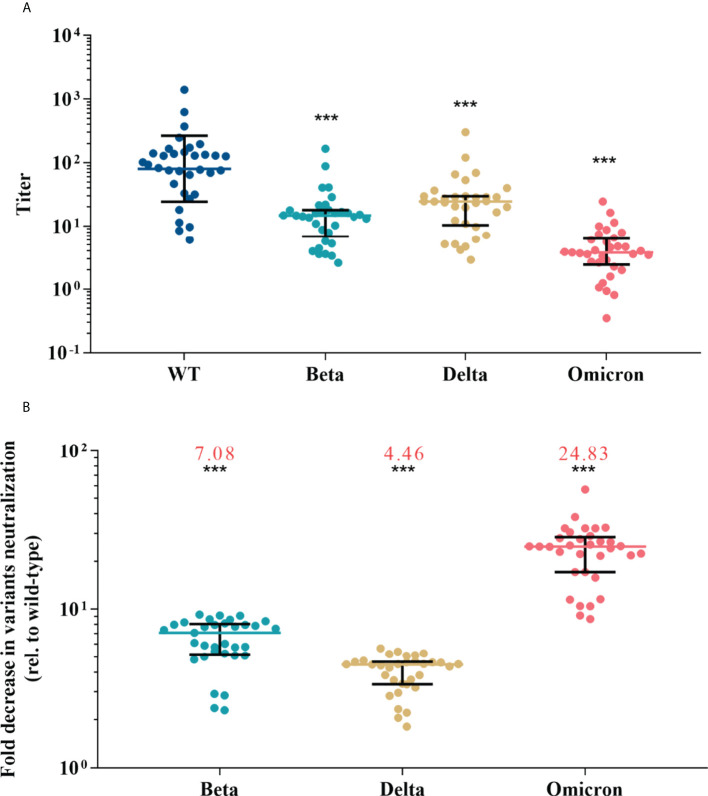
Neutralization responses against wild-type and variants of SARS-CoV-2 in convalescent COVID-19 patients. **(A)** Neutralization titers against wild-type and variants of SARS-CoV-2; colored bars represent geometric means and black bars represent geometric standard deviations. **(B)** Fold decrease in neutralization for each variant relative to wild-type virus, which was calculated by dividing IC50s titer of the SARS-CoV-2 variant by that of wild-type from the same vaccine sample; colored bars represent medians and black bars represent interquartile ranges; the median fold decrease in each variant neutralization post primary or booster vaccines is shown as a number in red. Statistical comparisons between groups were evaluated by Wilcoxon signed-ranked test. ***p<0.001 as compared with wild-type.

**Figure 3:**
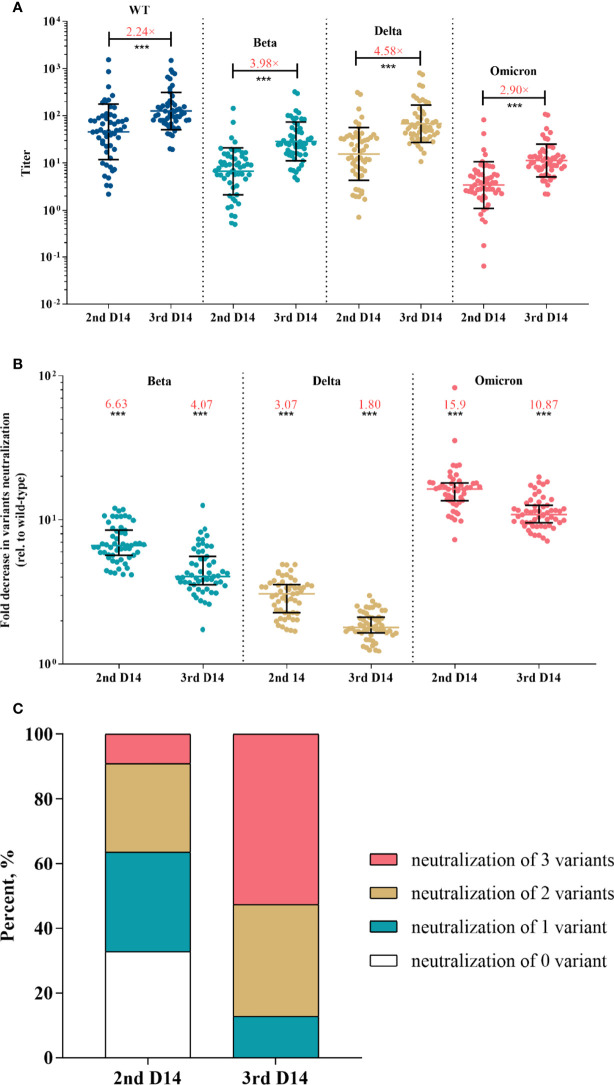
Neutralization responses against SARS-CoV-2 and variants in Coronavac recipients post primary and booster vaccinations. **(A)** Neutralization titers against wild-type and variants of SARS-CoV-2 post primary or booster vaccines; colored bars represent geometric means and black bars represent geometric standard deviations; the median fold-increase in neutralization titer post booster versus primary vaccine is shown as a number with “×” symbol in red; ***p<0.001 as compared with day 14 post the second dose. **(B)** Fold decrease in neutralization for each variant relative to wild-type virus, which was calculated by dividing IC50s titer of the SARS-CoV-2 variant by that of wild-type from the same vaccine sample; colored bars represent medians and black bars represent interquartile ranges; the median fold decrease in each variant neutralization post primary or booster vaccines is shown as a number in red; ***p<0.001 as compared with wild-type. **(C)** Neutralization breadth of variants elicited by primary and booster vaccination. Statistical comparisons between groups were evaluated by Wilcoxon signed-ranked test. 2nd D14, day 14 post the second dose; 3rd D14, day 14 post the third dose.

The results of pVNT showed that convalescent serum neutralized wild-type SARS-CoV-2 with a GMT of 80.12 (95% CI, 52.49-122.28), while the GMT against Beta, Delta, and Omicron declined to 13.06 (95% CI, 9.44-18.06), 20.50 (14.37-29.23), and 3.64 (2.66-4.97), respectively (p <0.001 for each variant) (**Figure 2A**). Compared with activity against the wild-type strain, serum neutralization efficiency was significantly reduced by a factor of 7.08 (IQR, 5.24-7.99) for Beta, 4.46 (IQR, 3.37-4.64) for Delta, and 24.83 (IQR, 17.15-28.08) for Omicron (p <0.001 for each variant) (**Figure 2B**). Among vaccine recipients, the neutralization titer against wild-type on day 28 post the first dose was low, reached a peak on day 14 post the second dose (GMT, 45.77; 95% CI, 31.74-66.02), and almost undetectable six to eight months after the second dose (**Figure S4**). The GMT against variants was 6.68 (95% CI, 4.90-9.11) for Beta, 15.54 (95% CI, 10.98-22.02) for Delta, and 3.42 (95% CI, 2.52-4.66) for Omicron (**Figure 3A**). The fold decrease of neutralization titer relative to wild-type was 6.63 (IQR, 5.68-8.41) for Beta, 3.07 (IQR, 2.29-3.55) for Delta, and 15.9 (IQR, 11.33-17.81) for Omicron (p <0.001 for each variant) (**Figure 3B**).

### Additional booster vaccine confers enhanced magnitude and breadth of variants neutralization

After the booster dose vaccination, neutralization titer against the wild-type strain increased with a median of 2.24-fold compared with the level post the primary shot (p <0.001) (**Figure 3A**). Strikingly, the booster dose obtained greater increases in variants neutralization with a median of 3.98-, 4.58- and 2.90-fold for Beta, Delta and Omicron, respectively (p <0.001 for each variant) (**Figure 3A**). The median fold decrease of variants neutralization was 4.07 (IQR, 3.61-5.48) for Beta, 1.80 (IQR, 1.66-2.12) for Delta, and 10.87 (IQR, 9.55-12.53) for Omicron (**Figure 3B**), which was significantly lower than that post the primary vaccination (p <0.001 for each variant), suggesting that the booster reduced variants escape from neutralizing.

Next, we investigated the neutralization breadth of variants. Results showed that the proportion of recipients able to neutralize Beta, Delta and Omicron increased from 36.36%, 67.27% and 9.09% post the primary vaccines to 87.27%, 100% and 52.73% post the booster, respectively. The proportion of recipients able to neutralize 2 or more variants increased from 36.36% post the primary vaccinations to 87.27% after the booster (**Figure 3C**). We also performed a multivariate regression analysis to explore the correlates of neutralization breadth of variants. Neutralization titer against wild-type SARS-CoV-2 was identified the strongest correlate of breadth (effect estimate 1.24 additional variants neutralized per log_10_ increase in neutralization titer, 95% CI, 0.89–1.59; adjusted p < 0.001) (**Table 2**). There was no significant impact of age, sex or BMI on neutralization breadth (p >0.1 for each variable).

**Table 2:** Effect estimates of multivariate regression analysis for each variable on neutralization breadth.

Variables	Effect estimate	95% CI	p-value
Neutralization titer against wild-type of SARS-CoV-2	1.24	0.89 to 1.59	<0.001
Age	0.03	-0.01 to 0.06	0.13
Sex	-0.05	-0.36 to 0.26	0.74
BMI	-0.05	-0.16 to 0.05	0.32

**Figure 4:**
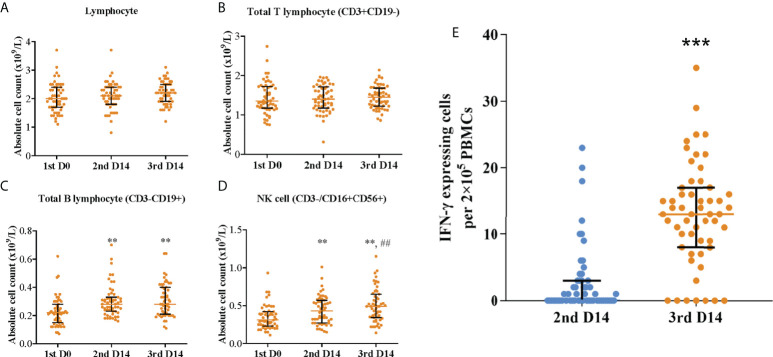
Blood lymphocyte subset distribution and specific T-cell responses post primary and booster vaccinations. Absolute count of **(A)** lymphocyte, **(B)** total T lymphocyte (CD3+CD19-), **(C)** total B lymphocyte (CD3-CD19+), and **(D)** NK cell (CD3-/CD16+CD56+); **p<0.01 as compared with day 0 post the first dose; ^##^p<0.01 as compared with day 14 post the second dose. **(E)** Specific T-cell responses; ***p<0.001 as compared with day 14 post the second dose. Colored bars represent medians and black bars represent interquartile ranges. Statistical comparisons between groups were evaluated by Wilcoxon signed-ranked test. 1st D0, day 0 post the first dose; 2nd D14, day 14 post the second dose; 3rd D14, day 14 post the third dose.

To assess the cellular immune responses elicited by the Coronavac vaccination, the blood lymphocyte subset distribution and the specific T-cell responses were also measured and correlated to neutralization titer and breadth. No significant difference was observed in lymphocyte subset distribution, except that B cells (CD3-CD19+) and NK cells (CD3-/CD16+CD56+) increased significantly in number and proportion post second and third dose vaccinations (**Figures 4A–D**, **S1** and **S2**). ELISpot assay showed that the median T-cell response level increased from 0 (IQR, 0-3) spot forming units per 2 × 10^5^ PBMCs post the primary vaccination to 13 (IQR, 8.5-16.5) after the booster (p <0.001) (**Figure 4E**). As expected, T-cell response level was robustly correlated with neutralization titer against the wild-type (Pearson R = 0.92; 95% CI, 0.87-0.95; p <0.001) and neutralization breadth of variants (Pearson R = 0.78; 95% CI, 0.70-0.85; p <0.001) (**Figure S5**). In contrast, B cells and NK cells were weakly correlated with neutralization titer against the wild-type and neutralization breadth of variants.

### Neutralization tier and breadth of SARS-CoV-2 variants can be predicted by anti-RBD IgG levels

**Figure 5:**
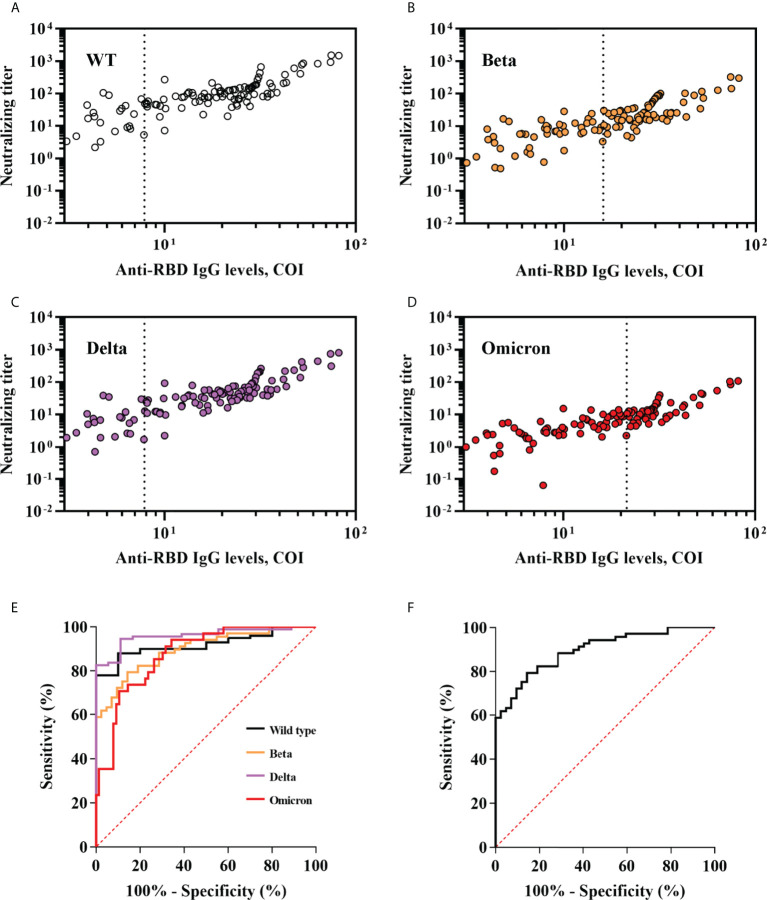
Performance of anti-RBD IgG levels in predicting neutralization titer and breadth of SARS-CoV-2 variants. **(A–D)** Anti-RBD IgG levels were plotted against neutralization of wild-type and variants of SARS-CoV-2. Optimal anti-RBD IgG cut-offs for predicting positive neutralization were determined by ROC analyses in **(E)** and are indicated with a vertical dashed line. **(E)** ROC analyses assessing the ability of anti-RBD IgG levels to predict neutralization titers against wild-type and variants of SARS-CoV-2. Positive neutralization titer was defined as ≥10. **(F)** ROC analyses assessing the ability of anti-RBD IgG levels to predict neutralization breadth. Broad neutralization breadth was defined as ≥2.

Anti-SARS-CoV-2 serological assays were easily accessible in clinical practice, whereas neutralization is not. Because RBD is crucial for viral entering and targeted by most of vaccines, we measured anti-RBD IgG level and correlated it to neutralization titer and breadth. We observed that anti-RBD IgG level was robustly correlated with the neutralization titer (Pearson R = 0.81, 95% CI, 0.73-0.86 for wild type; Pearson R = 0.81, 95% CI, 0.74-0.87 for Beta; Pearson R = 0.85, 95% CI, 0.78-0.89 for Delta; Pearson R = 0.81, 95% CI, 0.73-0.86 for Omicron; p <0.001 for wild type and each variant) and neutralization breadth (Pearson R = 0.77; 95% CI, 0.69-0.84; p < 0.001). ROC analyses showed that anti-RBD IgG level performed well at predicting positive neutralization of wild-type and variants (neutralization titer >10) and breadth (neutralization of ≥2 variants) (**Figure 5**). Area under curve (AUC) was 0.92, 0.90, 0.96 and 0.88 for wild-type, Beta, Delta and Omicron respectively, and 0.90 for breadth. The optimal cut-offs of COI were as follows: 7.84 achieved 88% sensitivity (Se) and 90% specificity (Sp) for wild-type; 16.93 achieved 79.41% Se and 85.71% Sp for Beta; 7.91 achieved 94.57% Se and 88.89% Sp for Delta; 27.34 achieved 70.59% Se and 89.47% Sp for Omicron; 16.93 achieved 79.41% Se and 85.71% Sp for breadth (**Figure 5**).

## Discussion

As most currently available SARS-CoV-2 vaccines were wild-type-based, it is critical to know the cross-neutralization against variants induced by vaccines. In this study, we assessed the neutralization magnitude and breadth of SARS-CoV-2 variants elicited by two-dose or three-dose of CoronaVac vaccines. We observed that second-dose waning was mitigated by a third dose vaccination that also increased and broadened neutralization of variants in the short term in longitudinally sampled participants. These findings may help evaluate the necessity and urgency to receive additional or even forward booster dose vaccination against current dominant variants like Omicron, or other potential SARS-CoV-2 variants in the future.

A growing body of studies had reported that current dominant variants especially Omicron were highly resistant to sera from convalescent or two-dose vaccine. Previous studies had found a significant reduction by a factor of 10 to 80 in neutralizing efficiency against Omicron using convalescent sera (25, 31, 32). A few studies reported that neutralization against Omicron was even undetectable in most of two-dose inactivated vaccine recipients (33, 34). Strikingly, a third homologous or heterologous booster vaccination following two doses of inactivated vaccines obtained more than 3-fold increase of neutralizing activity against wild-type and variants compared with that post the second one (21, 30, 35). Our findings are quite comparable with the previous ones. In the present study, more than half participants showed detectable neutralization against Omicron after receiving the third booster dose vaccination, while this proportion was less than 10% post the second dose. We observed that T-cell response level was robustly correlated with neutralization titer and breadth. It makes sense that T-cell responses were crucial for memory B cell production, antibody affinity maturation and the breadth of antigen recognition. Interestingly, total counts of B cells (CD3-CD19+) and NK cells (CD3-/CD16+CD56+) also increased by the booster vaccine in this study. One study reported that CD3-CD56+ NK cells frequency in the volunteers who recovered from mild COVID-19 (Mild CoV) presented a significant increase compared to the healthy control (HC) and individuals recovering from severe COVID-19 (Severe CoV) groups, indicating an essential role of NK cells in the process of SARS-CoV-2 control (36). However, protection value of NK cells elicited by vaccines and underlying mechanisms should be further elucidated.

Since the immunogen was wild-type-based for most currently available vaccines, it is essential to determine elicited neutralization breadth of variants especially for rapidly evolving virus like SARS-CoV-2. A recent study found that mRNA booster vaccine significantly enhanced variant neutralization breadth even in the subset of participants with poor pre-booster responses (37). Similarly, the present study showed that the booster achieved two or more variants neutralized in almost 90% of recipients, while this proportion was less than half post the primary vaccine. This substantial increase of breadth may be attributed to two possible reasons: (1) further affinity maturation of existing antibodies; (2) targeting new epitopes shared among variants. The first potential reason may be supported by the significant increase of neutralization titer against the wild-type and three variants post the booster vaccination. The second probable cause may be preliminarily evidenced by our results that the neutralization efficiency reduction for variants after the booster dose was less than that post the second one. These findings are consistent with another study reporting that three doses of BNT162b2 vaccine obtained significant lower neutralization efficiency reduction of variants than the primary vaccination (24). These implied that the booster may broaden recognized epitopes shared among variants, which need further demonstration.

In the present study, we observed that neutralization breadth significantly associated with neutralization titer against the wild-type. This result is in line with a previous study showing that the neutralization titer against the wild-type was the strongest correlate of breadth among mRNA and viral vectored vaccines recipients, with an effect estimate of 1.4 additional variants neutralized per log_10_ increase in neutralization titer (37). In that study, Naranbhai et al. also found that neutralization breadth was reduced with age which was not observed in the present study. This may because most of the participants enrolled in the present study were young adults. Given that neutralization is not easy to measure in clinical practice, it is important to find a surrogate. Interestingly, we found that neutralization titers and breadth can be well predicted by anti-RBD IgG level. Another two studies on mRNA vaccines corroborate this finding (37, 38). It makes sense because RBD is crucial for viral entering through interacting with the ACE2 receptor and targeted by most of currently available vaccines. This highlights the potential use of this easily accessible clinical assay in predicting SARS-CoV-2 and variants neutralization.

Our study has several limitations. First, as most of the enrolled participants were young adults, we were unable to investigate the impact of age on neutralization antibody response level. Another limitation of this study is that neutralization elicited by convalescent serum was estimated at only one time-point. Monitoring the immune response at serial time-points would help uncover the durability and breadth of neutralization antibody responses over time among convalescent COVID-19 patients.

In conclusion, while the dominant variants especially Omicron greatly escape from the neutralization elicited by previous infection and wild-type-based vaccines, additional homologous booster of Coronavac can restore breadth against current SARS-CoV-2 variants including Omicron. Additional or even forward booster shots were worth recommending as we await the next generation of vaccines.

## Data availability statement

The original contributions presented in the study are included in the article/**Supplementary Material**. Further inquiries can be directed to the corresponding author.

## Ethics statement

The studies involving human participants were reviewed and approved by Medical Ethical Committee of Sir Run Run Shaw Hospital. The patients/participants provided their written informed consent to participate in this study.

## Author contributions

LZ: Conceptualization, Data curation; Funding acquisition; Investigation; Validation; Roles/Writing - original draft. HC: Data curation; Investigation; Validation. SY: Data curation; Investigation. YZ, XS, XH, HY, DW, JL, and YW: Data curation; Investigation. SW: Conceptualization, Data curation; Funding acquisition; Investigation; Project administration; Validation; Roles/Writing - original draft. All authors contributed to the article and approved the submitted version.

## Funding

This study was funded by Department of Education of Zhejiang Province (Y202043596), Natural Science Foundation of Zhejiang province (LQ21H190005) and National Natural Science Foundation of China (82102476).

## Conflict of interest

The authors declare that the research was conducted in the absence of any commercial or financial relationships that could be construed as a potential conflict of interest.

## Publisher’s note

All claims expressed in this article are solely those of the authors and do not necessarily represent those of their affiliated organizations, or those of the publisher, the editors and the reviewers. Any product that may be evaluated in this article, or claim that may be made by its manufacturer, is not guaranteed or endorsed by the publisher.
