# Supplementary material for: The impact of CoronaVac on the neutralization breadth and magnitude of the antibody response to SARS-CoV-2 viruses
Source: Front Immunol. 2022 Sep 20;13:990071. doi: 10.3389/fimmu.2022.990071 (PMC9530635; doi:10.3389/fimmu.2022.990071)
Supplement: Supplementary file 1 [file DataSheet_1.docx]

Supplementary materials

**Safety outcomes**

The most common injection site adverse reaction was pain (47.27%), while the most common system adverse reaction was myalgia (29.09%) (Table S1).

Table S1. Solicited and unsolicited adverse reactions.

|  | **The second dose** | | | **The third dose** | | |
| --- | --- | --- | --- | --- | --- | --- |
|  | **Day 0** | **Day 14** | **Day 28** | **Day 0** | **Day 14** | **Day 28** |
| **Solicited Injection Site Adverse Reactions** | | | | | | |
| Any | 40 (72.73%) | 2 (3.64%) | 1 (1.82%) | 37 (67.27%) | 0 | 0 |
| Grade 1 | 38 (69.09%) | 2 (3.64%) | 1 (1.82%) | 35 (63.64%) | 0 | 0 |
| Grade 2 | 2 (3.64%) | 0 | 0 | 2 (3.64%) | 0 | 0 |
| Pain | 26 (47.27%) | 2 (3.64%) | 1 (1.82%) | 26 (47.27%) | 0 | 0 |
| Grade 1 | 25 (45.45%) | 2 (3.64%) | 1 (1.82%) | 25 (45.45%) | 0 | 0 |
| Grade 1 | 1 (1.82%) | 0 | 0 | 1 (1.82%) | 0 | 0 |
| Erythema | 1 (1.82%) | 0 | 0 | 1 (1.82%) | 0 | 0 |
| Grade 1 | 1 (1.82%) | 0 | 0 | 1 (1.82%) | 0 | 0 |
| Induration | 5 (9.09%) |  |  | 5 (9.09%) |  |  |
| Grade 1 | 4 (7.27%) | 0 | 0 | 4 (7.27%) | 0 | 0 |
| Grade 2 | 1 (1.82%) | 0 | 0 | 1 (1.82%) | 0 | 0 |
| Pruritus | 0 | 0 | 0 | 1 (1.82%) | 0 | 0 |
| Grade 1 | 0 | 0 | 0 | 1 (1.82%) | 0 | 0 |
| Swelling | 8 (14.55%) | 0 | 0 | 4 (7.27%) | 0 | 0 |
| Grade 1 | 8 (14.55%) | 0 | 0 | 4 (7.27%) | 0 | 0 |
| **Solicited Systematic Adverse Reactions** | | | | | | |
| Any | 19 (34.55%) | 1 (1.82%) | 0 | 21 (38.18%) | 0 | 0 |
| Grade 1 | 18 (32.73%) | 1 (1.82%) | 0 | 20 (36.36%) | 0 | 0 |
| Grade 2 | 1 (1.82%) | 0 | 0 | 1 (1.82%) | 0 | 0 |
| Fever | 1 (1.82%) | 0 | 0 | 1 (1.82%) | 0 | 0 |
| Grade 1 | 1 (1.82%) | 0 | 0 | 1 (1.82%) | 0 | 0 |
| Fatigue | 1 (1.82%) | 0 | 0 | 4 (7.27%) | 0 | 0 |
| Grade 1 | 1 (1.82%) | 0 | 0 | 4 (7.27%) | 0 | 0 |
| Myalgia | 16 (29.09%) | 1 (1.82%) | 0 | 15 (27.27%) | 0 | 0 |
| Grade 1 | 15 (27.27%) | 1 (1.82%) | 0 | 14 (25.45%) | 0 | 0 |
| Grade 2 | 1 (1.82%) | 0 | 0 | 1 (1.82%) | 0 | 0 |
| Rash | 0 | 0 | 0 | 0 | 0 | 0 |
| Grade 1 | 0 | 0 | 0 | 0 | 0 | 0 |
| Cough | 0 | 0 | 0 | 1 (1.82%) | 0 | 0 |
| Grade 1 | 0 | 0 | 0 | 1 (1.82%) | 0 | 0 |
| Anorexia | 0 | 0 | 0 | 0 | 0 | 0 |
| Arthralgia | 0 | 0 | 0 | 0 | 0 | 0 |
| Dyspnea | 0 | 0 | 0 | 0 | 0 | 0 |
| Nausea | 0 | 0 | 0 | 0 | 0 | 0 |
| Pharyngalgia | 0 | 0 | 0 | 0 | 0 | 0 |
| Syncope | 0 | 0 | 0 | 0 | 0 | 0 |
| Vertigo | 1 (1.82%) | 0 | 0 | 0 | 0 | 0 |
| Grade 1 | 1 (1.82%) | 0 | 0 | 0 | 0 | 0 |
| Vomiting | 0 | 0 | 0 | 0 | 0 | 0 |
| **Unsolicited Adverse reaction** | | | | | | |
| Sneezing | 0 | 0 | 0 | 2 (3.64%) | 0 | 0 |
| Grade 1 | 0 | 0 | 0 | 2 (3.64%) | 0 | 0 |
| Lethargy | 1 (1.82%) | 0 | 0 | 1 (1.82%) | 0 | 0 |
| Grade 1 | 1 (1.82%) | 0 | 0 | 1 (1.82%) | 0 | 0 |

**Clinical laboratory tests**

Blood lymphocyte subset distribution before vaccination, on day 14 post the second dose, and on day 14 post the third dose was analyzed using the flow cytometry system of FACSCanto II (Becton, Dickinson And Company, Franklin Lakes, US), which included absolute count of white blood cell, absolute count and proportion of lymphocyte, total T lymphocyte (CD3+CD19-), total B lymphocyte (CD3-CD19+), NK cell (CD3-/CD16+CD56+), T4 cell (CD3+CD4+), T8 cell (CD3+CD4+), CD3+CD4+CD8+ T cell, and T4/T8 ratio.

Overall, no significant difference was observed in lymphocyte subset distribution, except that B cells (CD3-CD19+) and NK cells (CD3-/CD16+CD56+) increased significantly in number and proportion post second and third dose vaccination (p<0.0001, supplementary Fig. S1 and S2).


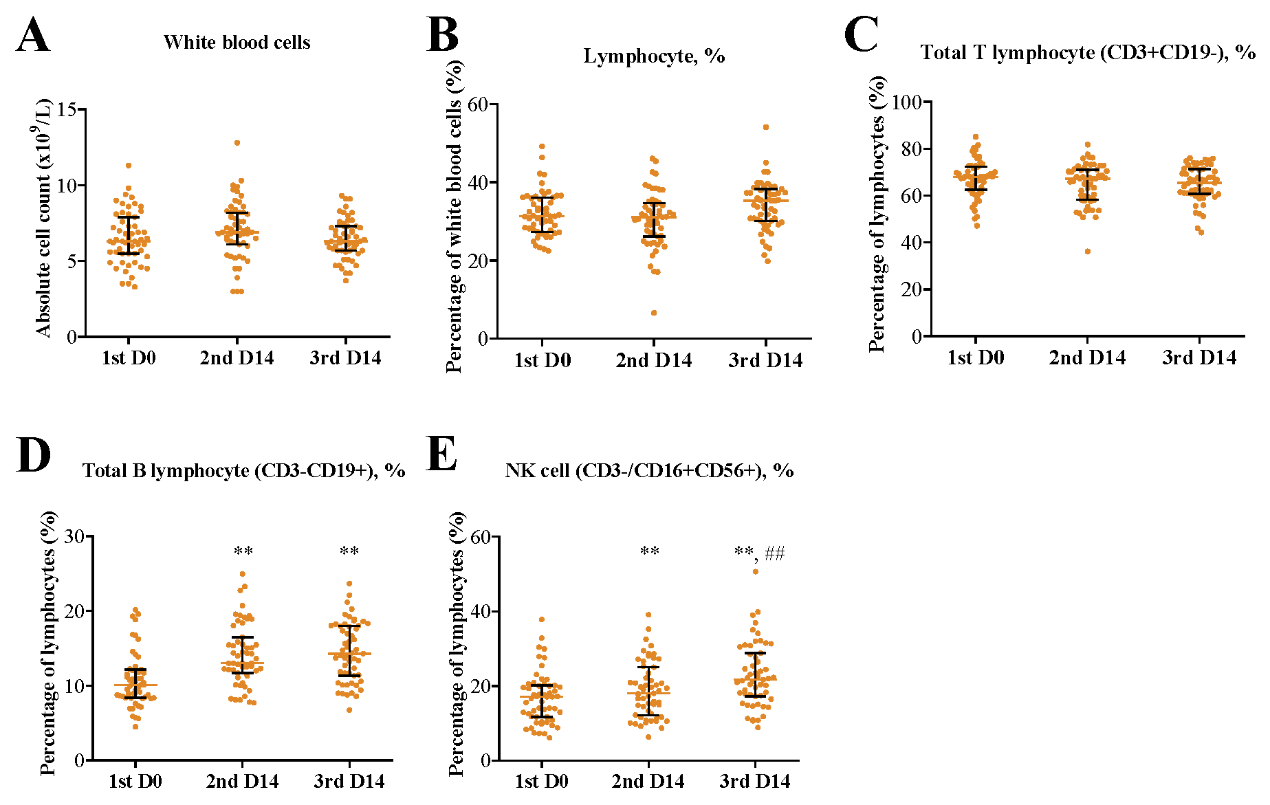


**Figure S1. Blood lymphocyte subset distribution.** (A) Absolute count of white blood cell. (B) Proportion of lymphocyte. (C) Proportion of total T lymphocyte (CD3+CD19-). (D) Proportion of total B lymphocyte (CD3-CD19+). (E) Proportion of NK cell (CD3-/CD16+CD56+). Level on day 14 post the second dose and third dose were compared with that before the first vaccination respectively, and level on day 14 post the third dose was also compared with that on day 14 post the second dose, using Wilcoxon signed-ranked test (**, p<0.01 as compared with 1st D0; ##, p<0.01 as compared with 2nd D14). 1st D0, day 0 post the first dose; 2nd D14, day D14 post the second dose; 3rd D14, day 14 post the third dose.


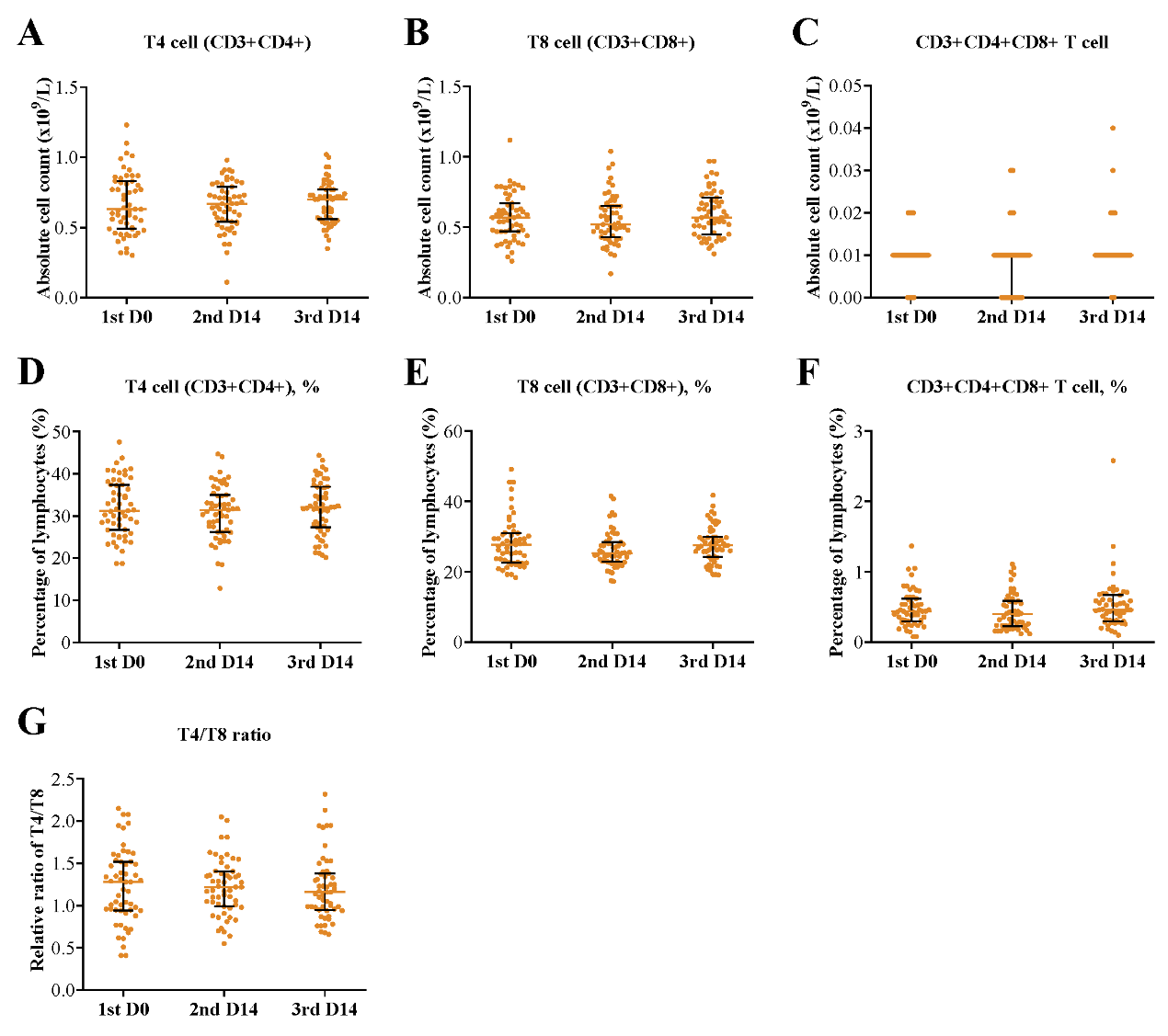


**Figure S2. Blood lymphocyte subset distribution of T lymphocyte.** (A and D) Absolute count and proportion of T4 cell (CD3+CD4+). (B and E) Absolute count and proportion of T8 cell (CD3+CD4+). (C and F) Absolute count and proportion of CD3+CD4+CD8+ T cell. (G) T4/T8 ratio. Level on day 14 post the second dose and third dose were compared with that before the first vaccination respectively, and level on day 14 post the third dose was also compared with that on day 14 post the second dose, using Wilcoxon signed-ranked test. 1st D0, day 0 post the first dose; 2nd D14, day 14 post the second dose; 3rd D14, day 14 post the third dose.


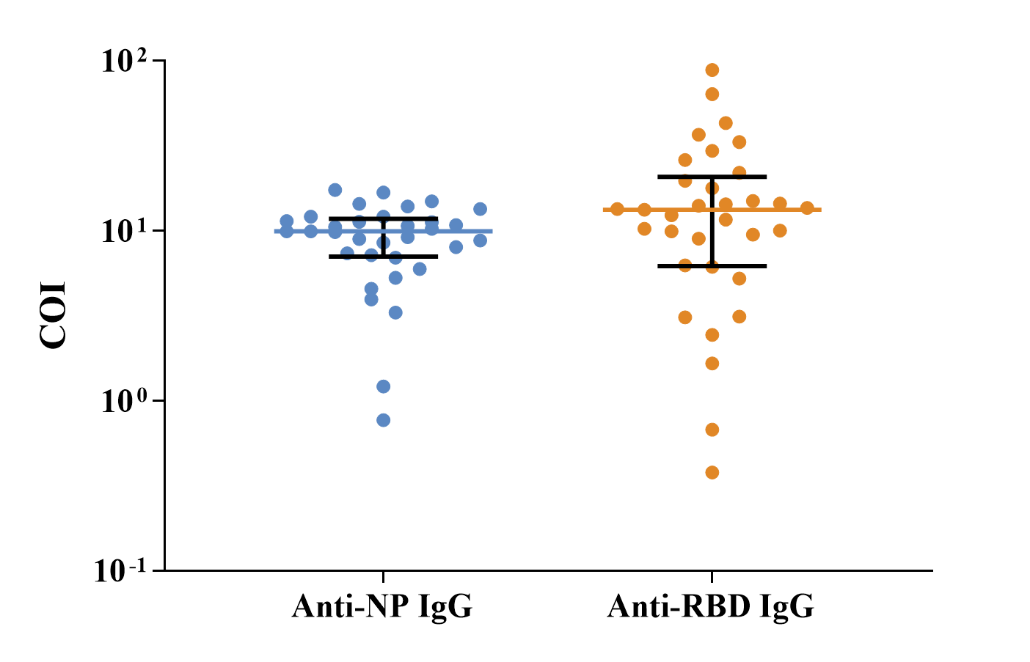


**Figure S3. Anti-NP and anti-RBD IgG levels in convalescent COVID-19 patients.** Colored bars represent medians and black bars represent interquartile ranges. Anti-NP IgG, anti-nucleocapsid protein IgG; anti-RBD IgG, anti-receptor binding domain IgG.


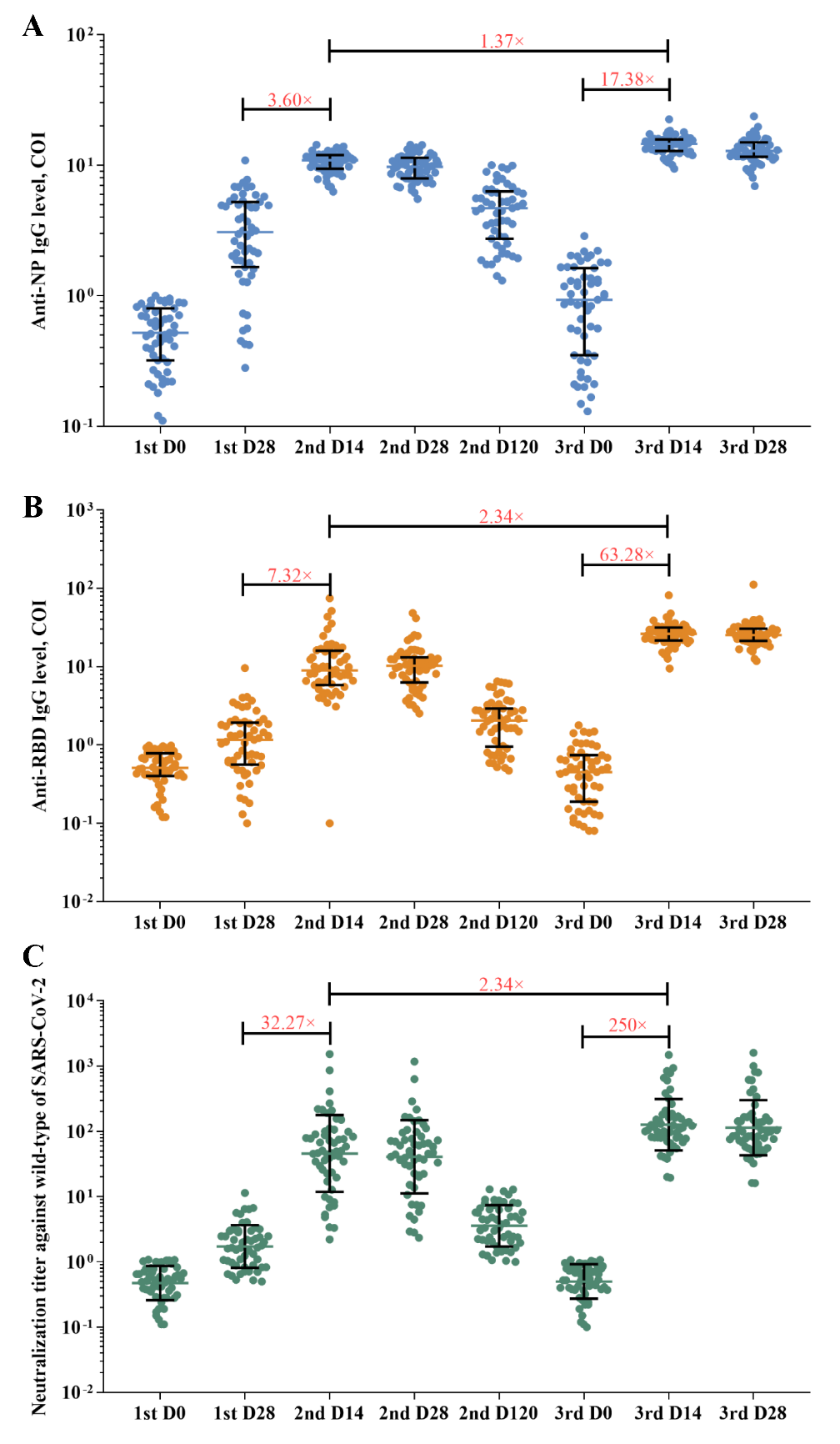


**Figure S4. Immune response dynamics in CoronaVac recipients.** (A) Anti-NP IgG levels; (B) Anti-RBD IgG levels; (C) Neutralizing titers against wild-type of SARS-CoV-2. The median fold-increase is shown as a number with “×” symbol in red. Anti-NP IgG, anti-nucleocapsid protein IgG; anti-RBD IgG, anti-receptor binding domain IgG.


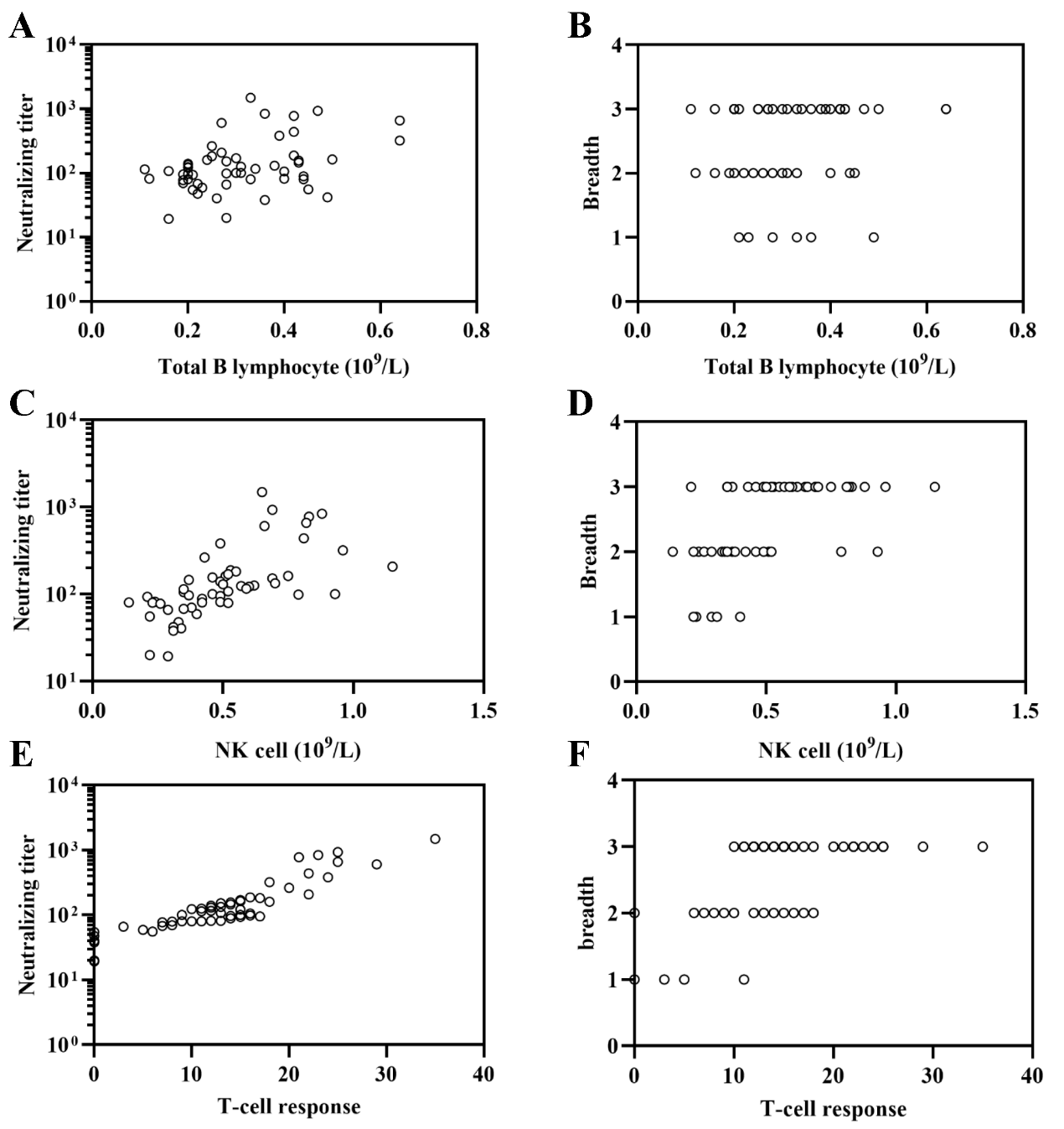


**Figure S5. Correlations between lymphocyte subsets and neutralization of wild-type and breadth.** (A-B) Total B lymphocytes were plotted against neutralization of wild-type and breadth; (C-D) NK cells were plotted against neutralization of wild-type and breadth; (E-F) T-cell response levels were plotted against neutralization of wild-type and breadth.
